# Investigating the Relationship between Telomere-Related Gene Variants and Leukocyte Telomere Length in Optic Neuritis Patients

**DOI:** 10.3390/jcm13092694

**Published:** 2024-05-03

**Authors:** Monika Duseikaite, Greta Gedvilaite, Paulius Mikuzis, Juste Andrulionyte, Loresa Kriauciuniene, Rasa Liutkeviciene

**Affiliations:** 1 Laboratory of Ophthalmology, Institute of Neuroscience, Lithuanian University of Health Sciences, Eivenių Street 2, LT-50161 Kaunas, Lithuania; greta.gedvilaite@lsmuni.lt (G.G.); loresa.kriauciuniene@lsmu.lt (L.K.); rasa.liutkeviciene@lsmu.lt (R.L.); 2 Faculty of Pharmacy, Lithuanian University of Health Sciences, Sukilėlių Pr. 13, LT-50166 Kaunas, Lithuania; 3 Medical Faculty, Lithuanian University of Health Sciences, LT-50161 Kaunas, Lithuania; paulius.mikuzis@stud.lsmuni.lt (P.M.); juste.andrulionyte@stud.lsmuni.lt (J.A.)

**Keywords:** optic neuritis, *TERF1*, *TERF2*, telomeres

## Abstract

Optic neuritis (ON) is a condition marked by optic nerve inflammation due to various potential triggers. Research indicates a link between telomeres and inflammation, as studies demonstrate that inflammation can lead to increased telomere shortening. **Aim**: We aimed to determine the associations of telomere-related telomeric repeat binding factor 1 (*TERF1*) rs1545827, rs10107605, and telomeric repeat binding factor 2 *(TERF2*) rs251796 polymorphisms and relative leukocyte telomere length (LTL) with the occurrence of ON. **Methods**: In this research, a total of 73 individuals diagnosed with optic neuritis (ON) were studied and the control group included 170 individuals without any health issues. The DNA samples were obtained from peripheral blood leukocytes, which were purified using the DNA salting-out technique. Real-time polymerase chain reaction (RT-PCR) assessed single-nucleotide polymorphisms (SNPs) and relative leukocyte telomere lengths (LTL). The data obtained were processed and analyzed using the “IBM SPSS Statistics 29.0” program. **Results**: Our study revealed the following results: in the male group, *TERF2* rs251796 (AA, AG, and TT) statistically significantly differed between the long and short telomere group, with frequencies of 65.7%, 22.9%, and 2.0% in long telomeres, compared to 35.1%, 56.8%, and 8.1% in the short telomere group (*p* = 0.013). The *TERF2* rs251796 CT genotype, compared to CC, under the codominant genetic model, was associated with 4.7-fold decreased odds of telomere shortening (*p* = 0.005). Meanwhile, CT+TT genotypes, compared to CC under the dominant genetic model, were associated with 3.5-fold decreased odds of telomere shortening (*p* = 0.011). Also, the CT genotype, compared to CC+TT, under the overdominant genetic model, was associated with 4.4-fold decreased odds of telomere shortening (*p* = 0.004). **Conclusions**: The current evidence may suggest a protective role of *TERF2* rs251796 in the occurrence of ON in men.

## 1. Introduction

Optic neuritis (ON) is the most common cause of subacute optic neuropathy in young adults [1]. ON, a demyelinating disorder characterized by acute, transient, predominantly monocular vision loss, is intricately linked to multiple sclerosis (MS), serving as the initial manifestation of MS in approximately 15–20% of affected individuals [2]. The occurrence of ON varies between 1 and 5 cases per 100,000 people [3], and its underlying mechanisms are not fully understood. Evidence suggests it is likely an immune-related condition, as indicated by the presence of systemic T-cells during disease onset and the detection of B-cells targeting myelin basic protein in the cerebrospinal fluid of individuals with ON [4].

Studies conducted globally indicate that the prevalence of the disease typically manifests within the age range of 18 to 45 years [5]. Etiologically, ON can be divided into two main forms, typical and atypical [6].

While the aforementioned factors are undeniably linked to optic nerve inflammation, the significance of genetic components remains uncertain, with growing acknowledgment of the role of hereditary or congenital influences on the onset of specific diseases.

Telomeres, consisting of repetitive nucleotide sequences, exhibit a high degree of conservation and comprise both double-stranded and single-stranded segments, which, when bound with shelterin proteins, protect chromosomal ends, thereby preserving genomic stability [7]. The shortening of telomeres with advancing age is a recognized phenomenon, with gradual diminishment contributing to somatic cell aging, programmed cell death, or potentially carcinogenic alterations, all of which can impact an individual’s health and life expectancy [8]. The telomeres, structured as histone octamer-composed nucleosomes, are stabilized through specific protein–protein and protein–DNA interactions between shelterin subunits and tandem repeat sequences [9]. This shelterin complex serves to protect chromosomes from end-joining and damage by forming unique T-loop structures [10]. The T-loop formations serve as protective caps at the chromosome ends, shielding them from being identified as breaks within the double-stranded DNA structure, thus maintaining the chromosomes’ stability and structural integrity [11]. Telomeric repeat binding factor 1 (TERF1) and telomeric repeat binding factor 2 (TERF2) act as inhibitors of telomerase activity, thereby participating in the control of telomere length [12]. Increased expression of TERF1 and TERF2 leads to the suppression of telomere length and effective telomere replication, potentially resulting in telomere shortening. Conversely, decreased expression may contribute to telomere elongation [13].

Chronic systemic inflammation is a recognized risk factor for various chronic disorders, including cardiovascular diseases, neurodegenerative diseases, autoimmune diseases, and cancers [14]. Numerous molecules known as inflammatory cytokines, such as tumour-necrosis factor-α (TNF-α), IL-1, IL-6 and many others, are produced by various cell types, particularly by macrophages and mast cells. These cytokines have several roles in the inflammatory process, including activation of the endothelium and leukocytes, as well as initiation of the acute-phase response [15]. Previous studies have indicated the relationship between telomere/telomerase dysfunction and inflammatory signaling [16]. Dysregulation of telomere-related genes and dysfunction in telomeres within diseases may lead to persistent, chronic, low-grade inflammation [17]. An interaction exists between inflammation and telomeres, as research indicates that inflammation accelerates telomere shortening, resulting in telomere dysfunction. At the same time, components of telomeres also contribute to the regulation of inflammatory responses [18].

As changes in telomere length are closely related to inflammation, we aimed to investigate whether telomere-associated genes are associated with the occurrence of optic neuritis.

## 2. Materials and Methods

The research was carried out at the Laboratory of Ophthalmology, Lithuanian University of Health Sciences, with approval from the Kaunas Regional Biomedical Research Ethics Committee (approval number: BE-2-102). Prior to participation, all individuals provided informed consent through a formal Informed Consent Form.

### 2.1. Study Group

The study included 243 subjects divided into two groups, a reference group (*n* = 170) and patients with ON (*n* = 73). The reference group was adjusted by sex and age to the ON group (*p* = 0.100 and *p* = 0.940, respectively). Relative leukocyte telomere length (LTL) was determined in all study subjects. There were no statistically significant differences between the reference and optical neuritis groups between relative LTL (*p* = 0.242). The demographic data of the study subjects are presented in Table 1.

### 2.2. DNA Extraction and Genotyping

Peripheral venous blood leukocytes were used for the extraction of genomic DNA using the salting-out technique. The analysis of single nucleotide polymorphisms (SNPs) of *TERF1* (rs1545827 and rs10107605) and *TERF2* (rs251796) was conducted using the real-time polymerase chain reaction (RT-PCR) method. TaqMan^®^ Genotyping assays (Applied Biosystems; Thermo Fisher Scientific, Inc., Waltham, MA, USA) were used according to the manufacturer’s protocols by StepOne Plus (Applied Biosystems) to determine SNPs. The assays IDs were C___1869846_10, C___1869856_10, and C___706068_10.

### 2.3. Relative Leukocyte Telomere Length Measurement

The relative leukocyte telomere length (LTL) was measured using the quantitative real-time PCR method developed by Cawthon (2002) [19]. This involved the analysis of telomeric DNA fragments and the reference gene albumin in duplicate. The RT-PCR analysis to determine relative LTL was conducted using a Rotor-Gene Q quantitative PCR machine (QIAGEN, Hilden, Germany). A reference DNA sample from the same age group was utilized for comparison. Additionally, DNA extracted from the commercial human cell line 1301 with extended telomeres (Sigma Aldrich, St. Louis, MO, USA) that served as a positive control. The primers used were obtained from IDT (Integrated DNA Technologies, Inc., Coralville, IA, USA) and were previously described in detail in our previous study [20].

In our study, we adopted a relative quantitative data analysis approach based on the recommendation by BioRad Laboratories in 2006. To determine the relative leukocyte telomere length (LTL) in peripheral blood leukocytes, we followed the relative analysis method developed by Livak in 2001 (relative LTL = 2^−ΔΔCt^) [21], after validating the PCR amplification efficiency. As specified by BioRad Laboratories in 2006, this approach is appropriate when the amplification efficiency of both telomere fragments and the albumin gene is between 90% and 105%, and the variation in efficiency between them is within 5%.

The ΔCt value for each sample is computed by finding the disparity between the Ct value of the tested telomere fragments and the Ct value of the reference albumin gene using the following equation:

ΔCt = Ct (telomere fragments) − Ct (reference albumin gene)

The ΔΔCt value characterizes the distinction between the ΔCt value of the test sample and the ΔCt value of the reference sample, which, similar to the test samples, has a concentration of 20 ng/μL and can be calculated using the following equation:

ΔΔCt = Ct (test sample) − Ct (reference sample) [22].

### 2.4. Statistical Analysis

Statistical analysis was performed using the “IBM SPSS Statistics 29.0” software (Statistical Package for the Social Sciences for Windows, Inc., Chicago, IL, USA). The data are presented in absolute numbers (percentages), median values, and interquartile ranges (IQRs). The demographic characteristics data were compared between the reference and ON groups using the Mann–Whitney U test. The frequencies of *TERF1* rs1545827 and rs10107605 and *TERF2* rs251796 genotypes and alleles are presented in percentages. Binary logistic regression analysis was conducted to assess the relationships between the chosen SNPs and the occurrence of ON. Various inheritance models and genotype combinations (including codominant, dominant, recessive, overdominant, and additive genetic models) were considered, giving an OR with a 95% confidence interval (CI). The Akaike Information Criterion (AIC) was used to identify the most suitable inheritance model, with the model having the lowest AIC value considered the best fit. A nonparametric Mann–Whitney U test compared different groups when the data distribution was not normal. Statistical significance was proven when the *p*-value was less than 0.05, indicating significant differences and correlations.

### 2.5. Limitations

Although our research offers valuable insights into telomere dynamics, it is important to acknowledge several limitations. Firstly, despite the participants’ age and sex, factors such as smoking, obesity, and stress disorders, which are known to affect telomere length, were not specifically analyzed in our study, leaving the influence of these variables on telomere attrition unexplored. Secondly, despite the fact ON is considered a rare disease, the sample size could be increased. In future studies, it would be beneficial to conduct comprehensive evaluations of lifestyle factors in order to fully understand the complex relationship between environmental influences and the dynamics of telomeres. One more limitation of our study is that the population under investigation appears to be predominantly composed of patients from Lithuania. This homogeneity may not fully reflect the genetic diversity, environmental exposures, and healthcare practices present in the broader global population. Variations in these factors across different geographic regions could potentially influence the associations observed between the *TERF1* and *TERF2* variants and the risk of ON. Therefore, the generalizability of our findings to other populations may be limited, and caution should be exercised when extrapolating the results to diverse ethnic or geographical groups. Despite these limitations, our study highlights the necessity for further research to gain a comprehensive understanding of telomere regulation and its implications for health and disease.

## 3. Results

The frequencies of genotypes and alleles for the single-nucleotide polymorphisms (SNPs) *TERF1* rs1545827, rs10107605 and *TERF2* rs251796 were analyzed within the study groups. No significant differences were found in the distribution of genotypes and alleles between patients diagnosed with ON and the reference group for the following SNPs: *TERF1* rs1545827, rs10107605, and *TERF2* rs251796 (see Supplementary Material Table S1).

The results of the Hardy–Weinberg equilibrium (HWE) test indicated that the genotypes of *TERF1* rs1545827 and *TERF2* rs251796 in the reference group did not show any significant deviation from HWE (*p* > 0.05) (Table 2). However, we identified that *TERF1* rs10107605 is not in HWE (Table 2). Regarding these findings, we excluded this SNP from the following analysis [23].

Binary logistic regression analysis was conducted in patients with ON and the reference group to investigate the associations of *TERF1* rs1545827, and *TERF2* rs251796 with ON occurrence. However, no statistically significant results were found when analyzing associations between ON occurrence and *TERF1* rs1545827 and *TERF2* rs251796 (Supplementary Material Table S2).

The frequencies of genotypes and alleles for the selected SNPs were analyzed within the study groups, stratified by sex. However, there were no statistically significant differences in the distribution of genotypes and alleles between females and males with ON and the reference group for the following SNPs (Supplementary Material Tables S3 and S5).

Binary logistic regression analysis was conducted in patients with ON and the reference group to investigate the associations of selected SNPs with ON occurrence in females and males. However, no statistically significant results were found when analyzing associations between ON occurrence in females and males and *TERF1* rs1545827 and *TERF2* rs251796 (Supplementary material Tables S4 and S6).

The frequencies of genotypes and alleles for the selected SNPs were analyzed within the study groups, stratified by age median. There were no statistically significant differences in the distribution of genotypes and alleles and binary logistic regression analysis between patients with ON and the reference group for the following SNPs: *TERF1* rs1545827 and *TERF2* rs251796 (age ≤ 30) (Supplementary Material Tables S7 and S8). The same results were found within the study groups when the study’s subjects’ age was over 30 years old (all *p* > 0.05) (Supplementary Material Tables S9 and S10).

Relative LTL was measured for 73 patients with ON and 170 reference group subjects. We found no statistically significant difference in relative LTL between the ON group and the reference group (median (IQR): 0.550 (0.698) vs. 0.518 (0.577), *p* = 0.242).

Regarding the median length of the reference groups relative LTL, we performed an analysis for subjects with long telomeres (when relative LTL ≥ 0.517) and those with short telomeres (when relative LTL < 0.517). However, no statistically significant differences in the frequencies of genotypes and alleles and binary logistic regression analysis for the selected SNPs were observed between the long and short telomeres (Supplementary Material Tables S11 and S12).

The frequencies of genotypes and alleles for the selected SNPs were analyzed within the study groups, stratified by sex regarding the median length of the reference groups relative LTL. However, there were no statistically significant differences in the distribution of genotypes and alleles and binary logistic regression analysis between females with ON and the reference group for the following SNPs (Supplementary Material Tables S13 and S14). When analyzing the male group, we observed that *TERF2* rs251796 (AA, AG, and TT) statistically significantly differed between the long and short group telomeres in males, with frequencies of 65.7%, 22.9%, and 2.0% in long telomeres, compared to 35.1%, 56.8%, and 8.1% in the short telomere group (*p* = 0.013) (Table 3).

Binary logistic regression analysis was conducted in males according to telomere shortening. The results revealed the following associations: the *TERF2* rs251796 AG genotype, compared to AA, under the codominant genetic model, was associated with 4.7-fold decreased odds of telomere shortening (OR: 0.215; 95% CI: 0.075–0.622; *p* = 0.005). Also, the AG+GG genotype, compared to AA, under the dominant genetic model, is associated with 3.5-fold decreased odds of telomere shortening (OR: 0.283; 95% CI: 0.107–0.746; *p* = 0.011). Lastly, the AG genotype compared to AA+GG, under the overdominant genetic model, is associated with 4.4-fold decreased odds of telomere shortening (OR: 0.226; 95% CI: 0.081–0.628; *p* = 0.004). The results are shown in Table 4.

The frequencies of genotypes and alleles for the selected SNPs were analyzed within the study groups, stratified by age median regarding the median length of the reference groups relative LTL. However, there were no statistically significant differences in the distribution of genotypes and alleles and binary logistic regression analysis between patients with ON and the reference group for the following SNPs (Supplementary Material Tables S15–S18).

## 4. Discussion

Our study aimed to explore the potential association between genetic variations in telomere maintenance-related genes, telomere length, and ON incidence. To achieve this, we conducted an analysis focusing on the following two polymorphisms located within genes associated with telomerase: *TERF1* rs1545827 and *TERF2* rs251796.

The results were evaluated according to gender and age groups. The relationship between relative LTL and age is the most studied and evidence-based in the literature; it is known that telomeres become shorter with years, and this is related to cellular aging mechanisms [24]. According to the results of our investigation, the frequencies of genotypes and alleles for the selected SNPs were analyzed within the study groups, stratified by age median. There were no statistically significant differences in the distribution of genotypes and alleles and binary logistic regression analysis between patients with ON and the reference group for the following SNPs: *TERF1* rs1545827 and *TERF2* rs251796 (age ≤ 30). The same results were found within the study groups when the study’s subjects’ age was over 30 years old (all *p* > 0.05). Also, the frequencies of genotypes and alleles for the selected SNPs were analyzed within the study groups, stratified by age median regarding the median length of the reference groups relative LTL. However, there were no statistically significant differences in the distribution of genotypes and alleles and binary logistic regression analysis between patients with ON and the reference group for the following SNPs.

Not only does relative LTL rely on age, but it also depends on genetic and environmental factors. Hereditary factors determine about 70% of relative LTL [25]; a sex difference has also been observed in many studies; they determined that women’s telomeres are statistically significantly longer than men’s [26]. Optic neuritis tends to affect females more than males, with a ratio of around 3:1, regardless of whether individuals are of Caucasian or Oriental descent. Despite this observation, the precise underlying mechanisms remain unclear. It is hypothesized that there may be a sex-specific susceptibility to autoimmune diseases, although further research is needed to clarify this relationship [27]. Kim et al. found that ON onset is later in men than in women (average 49 vs. 41 years of age), neuritic attacks are less frequent at onset (17 vs. 44%) and are less frequent during the course of the disease (0.08 vs. 0.27 per year) in men than in women. Males more often tend to develop an isolated form of myelitis (67 vs. 28%). Visual evoked potential testing showed a shorter latency of P100 in men. Men were also noted to have fewer acute optic neuritis attacks, independent of age at onset of the disease [28].

Bekaert and others reported that telomere attrition in their study proceeded faster in men (30.0 bp per year, R^2^ = 0.062) compared to women (20.3 bp per year, R^2^ = 0.028) [29]. During our investigation, the analysis showed that when analyzing the male group, the *TERF2* rs251796 AG genotype, compared to AA, under the codominant genetic model, is associated with 4.7-fold decreased odds of telomere shortening (OR: 0.215; 95% CI: 0.075–0.622; *p* = 0.005). The AG+GG genotype, compared to AA, under the dominant genetic model, is associated with 3.5-fold decreased odds of telomere shortening (OR: 0.283; 95% CI: 0.107–0.746; *p* = 0.011). Lastly, the AG genotype, compared to AA+GG, under the overdominant genetic model, is associated with 4.4-fold decreased odds of telomere shortening (OR: 0.226; 95% CI: 0.081–0.628; *p* = 0.004). The current evidence may suggest a protective role of *TERF2* rs251796 SNP in the occurrence of ON in men. We did not find any statistical significance in the distribution of genotypes and alleles and binary logistic regression analysis between females with ON and the reference group. These results could be due to the small sample size of the women studied and the characteristics of the population. According to The Optic Neuritis Treatment Trial, the majority (77%) of patients with ON are young women. Women are at least twice as likely to develop MS compared with men, yet the latter have been reported to have worse clinical outcomes and faster disease progression [30]. Several factors have been proposed to account for gender differences in MS including sex hormones, genetics, immune biases, and environmental influences [31]. Estrogen, as a sex hormone, has demonstrated efficacy in reducing the severity of experimental autoimmune encephalomyelitis in animal models of central nervous system (CNS) inflammation, as well as in clinical settings where treatment with estrogen derivatives correlated with decreased MRI-measured gadolinium-enhancing lesions in women with relapsing-remitting multiple sclerosis (RRMS) [32]. Additionally, estrogen has exhibited the ability to enhance retinal blood flow and protect the retinal nerve fiber layer (RNFL) in both animal and clinical models of optic nerve injury [33]. In a study involving an experimental model of Leber hereditary optic neuropathy, 17β-estradiol activated mitochondrial biogenesis and improved energetic competence, leading researchers to theorize that the protective effects of estrogen could explain the higher prevalence of Leber hereditary optic neuropathy in men compared to women [34]. While it is plausible that estrogen’s neuroprotective benefits may have contributed to the relatively preserved RNFL values observed in women compared to men in a particular study, specific evaluation of sex hormones was not conducted. Thus, further research is needed. Our study did not evaluate environmental factors such as smoking, psychological stress, diet, obesity, or physical activity, which could affect the telomere length in the patients of ON and healthy subjects [35]. In future studies, it would be beneficial to conduct in-depth assessments of lifestyle factors to interpret the relationship between environmental factors and the dynamics of telomeres.

To our knowledge, no studies are currently examining the relative LTL associations with the development of ON. However, several studies have investigated the relationship between MS and relative LTL. In the case of MS, it has been suggested that the reduced telomere length observed in patients with primary progressive MS represents the most advanced stage of the disease, indicating that biological aging contributes to MS progression. This association between shorter telomere length and increased disability and brain atrophy suggests a link between telomere length and disease severity [36]. Additionally, Krysko et al. reported that greater MS aggressiveness operates mechanisms that initiate specific DNA damage, which deactivates telomerase to prolong telomere length, leading to telomere shortening [37].

In the scenario of potential telomere shortening in the future, it remains ambiguous whether this is a precursor or an outcome of ON or MS development. If accelerated telomere shortening occurred before disease manifestation, it could become a risk factor. However, leukocyte telomere length could also indicate the combined effect of oxidative stress and inflammation throughout the progression, severity, and duration of the disease.

Many authors put forward the hypothesis that the shortening of telomeres in chronic inflammatory diseases may result from a prolonged and severe inflammatory process and oxidative stress [38,39,40,41]. New significant differences that could explain the changes in telomere length in ON inflammation were not found, but this work may contribute to the hypothesis previously put forward by other authors. However, further research is needed to comprehend the underlying mechanisms and their potential therapeutic implications.

## 5. Conclusions

To conclude, the current evidence may suggest a protective role of *TERF2* rs251796 in the occurrence of ON in men.

## Figures and Tables

**Table 1:** Demographic characteristics of the study.

Characteristics		Group		*p*-Value
		ON Group	Reference Group	
Sex	Males, N (%)	27 (37)	45 (26.5)	0.100
	Females, N (%)	46 (63)	125 (73.5)	
Age median (IQR)		33 (17)	29.5 (22)	0.940
Relative leukocyte telomere length median (IQR)		0.550 (0.698)	0.517 (0.577)	0.242

Mann–Whitney U test was used; ON—optical neuritis; IQR—interquartile range; *p*-value: significance level (alpha = 0.05).

**Table 2:** Analysis of Hardy–Weinberg equilibrium in the reference group.

Gene and SNP	Allele Frequencies		Genotype Distribution	*p*-Value
*TERF1* rs1545827	0.60 C	0.40 T	23/89/58	0.223
*TERF1* rs10107605	0.89 A	0.11 C	9/21/140	**<0.0001**
*TERF2* rs251796	0.70 A	0.30 G	18/66/86	0.324

SNP—single-nucleotide polymorphism; *p*-value—significance level (alpha = 0.05). Statistically significant results are marked in bold.

**Table 3:** Frequencies of genotypes and alleles of *TERF1* rs1545827, and *TERF2* rs251796 in the long and short telomere groups in males (T/S median = 0.517).

Gene, SNP	Genotype, Allele	Long Telomeres	Short Telomeres	*p*-Value
*TERF1* rs1545827	CC CT TT Total Allele C T	13 (37.1) 20 (57.1) 2 (5.7) 35 (100) 46 (65.7) 24 (34.3)	12 (32.4) 20 (54.1) 5 (13.5) 37 (100) 44 (59.5) 30 (40.5)	0.530 0.438
*TERF2* rs251796	AA AG GG Total Allele A G	23 (65.7) 8 (22.9) 4 (11.4) 35 (100) 54 (77.1) 16 (22.9)	13 (35.1) 21 (56.8) 3 (8.1) 37 (100) 47 (63.5) 27 (36.5)	**0.013** 0.074

*p*-value: significance level (alpha = 0.05); statistically significant results are marked in bold.

**Table 4:** Binary logistic regression analysis of *TERF1* rs1545827 and *TERF2* rs251796 in telomere shortening in males.

Model	Genotype/Allele	OR (95% CI)	*p*-Value	AIC
*TERF1* rs1545827				
Codominant	CT vs. CC TT vs. CC	0.923 (0.340–2.509) 0.369 (0.060–2.274)	0.875 0.283	102.445
Dominant	CT+TT vs. CC	0.812 (0.307–2.146)	0.675	101.582
Recessive	TT vs. CC+CT	0.388 (0.070–2.145)	0.278	100.470
Overdominant	CT vs. CC+TT	1.133 (0.447–2.874)	0.792	101.688
Additive	T	0.719 (0.337–1.533)	0.393	101.017
*TERF2* rs251796				
Codominant	AG vs. AA GG vs. AA	0.215 (0.075–0.622) 0.754 (0.146–3.901)	**0.005** 0.736	94.815
Dominant	AG+GG vs. AA	0.283 (0.107–0.746)	0.011	94.921
Recessive	GG vs. AA+AG	1.462 (0.303–7.058)	0.636	101.531
Overdominant	AG vs. AA+GG	0.226 (0.081–0.628)	0.004	92.927
Additive	G	0.521 (0.249–1.094)	0.085	98.624

OR: odds ratio; CI: confidence interval; *p*-value: significance level (alpha = 0.05); AIC: Akaike information criterion; statistically significant results are marked in bold.

## Data Availability

Data will be sent upon request.

## Supplementary Materials

The following supporting information can be downloaded at: https://www.mdpi.com/article/10.3390/jcm13092694/s1, Table S1: Genotype and allele frequencies of single-nucleotide polymorphisms (*TERF1* rs1545827, rs10107605 and *TERF2* rs251796) within ON and reference groups; Table S2: Binary logistic regression analysis within patients with ON and reference group subjects; Table S3: Genotype and allele frequencies of *TERF1* rs1545827 and *TERF2* rs251796 within ON and reference group females; Table S4: Binary logistic regression analysis within females with ON and reference group females; Table S5: Genotype and allele frequencies of *TERF1* rs1545827 and *TERF2* rs251796 within ON and reference group males; Table S6: Binary logistic regression analysis within males with ON and reference group males; Table S7: Genotype and allele frequencies of *TERF1* rs1545827 and *TERF2* rs251796 within ON and reference group subjects (age ≤ 30); Table S8: Binary logistic regression analysis within patients with ON and reference group subjects (age ≤ 30); Table S9: Genotype and allele frequencies of *TERF1* rs1545827 and *TERF2* rs251796 within ON and reference group subjects (age > 30); Table S10: Binary logistic regression analysis within patients with ON and reference group subjects (age > 30); Table S11: Frequencies of genotypes and alleles of *TERF1* rs1545827 and *TERF2* rs251796 in the long and short telomere groups (T/S median = 0.517); Table S12: Binary logistic regression analysis of *TERF1* rs1545827 and *TERF2* rs251796 in telomere shortening; Table S13: Frequencies of genotypes and alleles of *TERF1* rs1545827 and *TERF2* rs251796 in the long and short telomere groups for females (T/S median = 0.517); Table S14: Binary logistic regression analysis of *TERF1* rs1545827 and *TERF2* rs251796 in telomere shortening in females; Table S15: Frequencies of genotypes and alleles of *TERF1* rs1545827 and *TERF2* rs251796 in the long and short telomere groups for subjects aged ≤ 30 (T/S median = 0.517); Table S16: Binary logistic regression analysis of *TERF1* rs1545827 and *TERF2* rs251796 in telomere shortening for subjects aged ≤ 30; Table S17: Frequencies of genotypes and alleles of *TERF1* rs1545827 and *TERF2* rs251796 in the long and short telomere groups for subjects aged > 30 (T/S median = 0.517); Table S18: Binary logistic regression analysis of *TERF1* rs1545827 and *TERF2* rs251796 in telomere shortening for subjects aged > 30.
