# Supplementary material for: Investigating the Relationship between Telomere-Related Gene Variants and Leukocyte Telomere Length in Optic Neuritis Patients
Source: J Clin Med. 2024 May 3;13(9):2694. doi: 10.3390/jcm13092694 (PMC11084964; doi:10.3390/jcm13092694)
Supplement: Supplementary file 1 [file jcm-13-02694-s001.zip › jcm-2951340-supplementary.pdf]

Supplementary material

Table S1. Genotype and allele frequencies of single nucleotide polymorphisms (*TERF1* rs1545827, rs10107605 and *TERF2* rs251796) within ON and reference groups.

Gene	Genotype/Allele	ON group (n=73) N (%)	Control group (n=170) N (%)	p-value
***TERF1* (rs1545827)**	CC	25 (34.2)	58 (34.1)	0.851
	CT	40 (54.8)	89 (52.4)	
	TT	8 (11)	23 (13.5)	
	In total:	73 (100)	170 (100)	
	Allele:<br>C<br>T	 90 (61.6)<br>56 (38.4)	 205 (60.3)<br>135 (39.7)	0.780
***TERF1* (rs10107605)**	AA	59 (80.8)	140 (82.4)	0.063
	AC	14 (19.2)	21 (12.4)	
	CC	0 (0)	9 (5.3)	
	In total:	73 (100)	170 (100)	
	Allele:<br>A<br>C	 132 (90.4)<br>14 (9.6)	 301 (88.5)<br>39 (11.5)	0.541
***TERF2* (rs251796)**	AA	36 (49.3)	86 (50.6)	0.983
	AG	29 (39.7)	66 (38.8)	
	GG	8 (11)	18 (10.6)	
	In total:	73 (100)	170 (100)	
	Allele:<br>A<br>G	 101 (69.2)<br>45 (30.8)	 238 (70)<br>102 (30)	0.856

ON – optic neuritis; *p*-value: significance level (alpha = 0.05).

Table S2. Binary logistic regression analysis within patients with ON and reference group subjects.

***TERF1* (rs1545827):**				
**Model**	**Genotype/Allele**	**OR (95% CI)**	**p-value**	**AIC**
Codominant	CT *vs.* CC<br>TT *vs.* CC	1.043 (0.573-1.898)<br>0.807 (0.318-2.048)	0.891<br>0.652	300.720
Dominant	CT+TT *vs.* CC	0.994 (0.558-1.772)	0.994	299.049
Recessive	TT *vs.* CC+CT	0.787 (0.334-1.851)	0.583	298.738
Overdominant	CT *vs.* CC+TT	1.103 (0.636-1.913)	0.727	298.927
Additive	T	0.938 (0.615-1.431)	0.767	298.961
***TERF2* (rs251796):**				
**Model**	**Genotype/Allele**	**OR (95% CI)**	**p-value**	**AIC**
Codominant	AG *vs.* AA<br>GG *vs.* AA	1.050 (0.585-1.884)<br>1.062 (0.423-2.662)	0.871<br>0.898	301.016
Dominant	AG+GG *vs.* AA	1.052 (0.608-1.821)	0.856	299.016

Recessive	GG *vs.* AA+AG	1.039 (0.430-2.511)	0.932	299.042
Overdominant	AG *vs.* AA+GG	1.039 (0.593-1.820)	0.895	299.032
Additive	G	1.037 (0.691-1.556)	0.861	299.019

ON – Optical Neuritis; OR: odds ratio; CI: confidence interval; *p*-value: significance level (alpha = 0.05); AIC: Akaike information criterion; Statistically significant results marked in bold; The most robust genetic model underlined (selected based on the lowest AIC value).

Table S3. Genotype and allele frequencies of *TERF1* rs1545827 and *TERF2* rs251796 within ON and reference groups females.

Gene	Genotype/Allele	ON group (n=46) N (%)	Control group (n=125) N (%)	p-value
***TERF1* (rs1545827)**	CC	15 (32.6)	43 (34.4)	0.173
	CT	28 (60.9)	61 (48.8)	
	TT	3 (6.5)	21 (16.8)	
	In total:	46 (100)	125 (100)	
	Allele:<br>C<br>T	 58 (63)<br>34 (37)	 147 (58.8)<br>103 (41.2)	0.477
***TERF2* (rs251796)**	AA	25 (54.3)	61 (48.8)	0.748
	AG	17 (37)	49 (39.2)	
	GG	4 (8.7)	15 (12)	
	In total:	46 (100)	125 (100)	
	Allele:<br>A<br>G	 67 (72.8)<br>25 (27.2)	 171 (68.4)<br>79 (31.6)	0.430

ON – optic neuritis; *p*-value: significance level (alpha = 0.05).

Table S4. Binary logistic regression analysis within females with ON and reference group females.

***TERF1* (rs1545827):**				
**Model**	**Genotype/Allele**	**OR (95% CI)**	**p-value**	**AIC**
Codominant	CT *vs.* CC<br>TT *vs.* CC	1.316 (0.629-2.754)<br>0.410 (0.107-1.572)	0.466<br>0.193	199.253
Dominant	CT+TT *vs.* CC	1.084 (0.528-2.223)	0.826	201.087
Recessive	TT *vs.* CC+CT	0.346 (0.098-1.219)	0.099	197.776
Overdominant	CT *vs.* CC+TT	1.632 (0.820-3.248)	0.163	199.159
Additive	T	0.823 (0.491-1.378)	0.459	200.582
***TERF2* (rs251796):**				
**Model**	**Genotype/Allele**	**OR (95% CI)**	**p-value**	**AIC**
Codominant	AG *vs.* AA<br>GG *vs.* AA	0.847 (0.411-1.742)<br>0.651 (0.197-2.154)	0.651<br>0.482	202.541
Dominant	AG+GG *vs.* AA	0.801 (0.406-1.577)	0.520	200.721
Recessive	GG *vs.* AA+AG	0.698 (0.219-2.225)	0.544	200.747
Overdominant	AG *vs.* AA+GG	0.909 (0.452-1.828)	0.789	201.064

Additive	G	0.821 (0.493-1.369)	0.450	200.554

ON – Optical Neuritis; OR: odds ratio; CI: confidence interval; *p*-value: significance level (alpha = 0.05); AIC: Akaike information criterion; Statistically significant results marked in bold; The most robust genetic model underlined (selected based on the lowest AIC value).

Table S5. Genotype and allele frequencies of *TERF1* rs1545827 and *TERF2* rs251796 within ON and reference groups males.

**Gene**	**Genotype/Allele**	**ON group (n=27) N (%)**	**Control group (n=45) N (%)**	**p-value**
***TERF1* (rs1545827)**	CC	10 (37)	15 (33.3)	0.107
	CT	12 (44.4)	28 (62.2)	
	TT	5 (18.5)	2 (4.4)	
	In total:	27 (100)	45 (100)	
	Allele:			
	C	32 (59.3)	58 (64.4)	0.533
	T	22 (40.7)	32 (35.6)	
***TERF2* (rs251796)**	AA	11 (40.7)	25 (55.6)	0.354
	AG	12 (44.4)	17 (37.8)	
	GG	4 (14.8)	3 (6.7)	
	In total:	27 (100)	45 (100)	
	Allele:			
	A	34 (63)	67 (74.4)	0.144
	G	20 (37)	23 (25.6)	

ON – optic neuritis; *p*-value: significance level (alpha = 0.05).

Table S6. Binary logistic regression analysis within males with ON and reference group males.

***TERF1* (rs1545827):**				
**Model**	**Genotype/Allele**	**OR (95% CI)**	**p-value**	**AIC**
Codominant	CT *vs.* CC	0.643 (0.225-1.833)	0.408	94.896
	TT *vs.* CC	3.750 (0.605-23.252)	0.156	
Dominant	CT+TT *vs.* CC	0.850 (0.314-2.304)	0.749	97.163
Recessive	TT *vs.* CC+CT	4.886 (0.876-27.245)	0.070	93.577
Overdominant	CT *vs.* CC+TT	0.486 (0.184-1.280)	0.144	95.105
Additive	T	1.312 (0.605-2.848)	0.492	96.790
***TERF2* (rs251796):**				
**Model**	**Genotype/Allele**	**OR (95% CI)**	**p-value**	**AIC**
Codominant	AG *vs.* AA	1.604 (0.576-4.468)	0.366	97.213
	GG *vs.* AA	3.030 (0.578-15.880)	0.190	
Dominant	AG+GG *vs.* AA	1.818 (0.691-4.782)	0.226	95.777
Recessive	GG *vs.* AA+AG	2.435 (0.501-11.832)	0.270	96.034
Overdominant	AG *vs.* AA+GG	1.318 (0.500-3.473)	0.577	96.954
Additive	G	1.692 (0.816-3.507)	0.158	95.234

ON – Optical Neuritis; OR: odds ratio; CI: confidence interval; *p*-value: significance level (alpha = 0.05); AIC: Akaike information criterion; Statistically significant results marked in bold; The most robust genetic model underlined (selected based on the lowest AIC value).

Table S7. Genotype and allele frequencies of *TERF1* rs1545827 and *TERF2* rs251796 within ON and reference groups subjects (age≤30).

Gene	Genotype/Allele	ON group (n=33) N (%)	Control group (n=89) N (%)	p-value
***TERF1* (rs1545827)**	CC	15 (45.5)	31 (34.8)	0.373
	CT	17 (51.5)	50 (56.2)	
	TT	1 (3)	8 (9)	
	In total:	33 (100)	89 (100)	
	Allele:<br>C<br>T	 47 (71.2)<br>19 (28.8)	 112 (62.9)<br>66 (37.1)	0.227
***TERF2* (rs251796)**	AA	15 (45.5)	44 (49.4)	0.544
	AG	16 (48.5)	35 (39.3)	
	GG	2 (6.1)	10 (11.2)	
	In total:	33 (100)	89 (100)	
	Allele:<br>A<br>G	 46 (69.7)<br>20 (30.3)	 123 (69.1)<br>55 (30.9)	0.928

ON – optic neuritis; *p*-value: significance level (alpha = 0.05).

Table S8. Binary logistic regression analysis within patients with ON and reference group subjects (age≤30).

Model	Genotype/Allele	OR (95% CI)	p-value	AIC
***TERF1* (rs1545827):**				
**Model**	**Genotype/Allele**	**OR (95% CI)**	**p-value**	**AIC**
Codominant	CT *vs.* CC<br>TT *vs.* CC	0.703 (0.308-1.606)<br>0.258 (0.030-2.259)	0.403<br>0.221	144.263
Dominant	CT+TT *vs.* CC	0.641 (0.285-1.445)	0.284	143.293
Recessive	TT *vs.* CC+CT	0.316 (0.038-2.633)	0.287	142.961
Overdominant	CT *vs.* CC+TT	0.829 (0.372-1.846)	0.646	144.223
Additive	T	0.622 (0.312-1.240)	0.177	142.558
***TERF2* (rs251796):**				
**Model**	**Genotype/Allele**	**OR (95% CI)**	**p-value**	**AIC**
Codominant	AG *vs.* AA<br>GG *vs.* AA	1.341 (0.583-3.083)<br>0.587 (0.115-2.986)	0.490<br>0.521	145.162
Dominant	AG+GG *vs.* AA	1.173 (0.526-2.615)	0.696	144.281
Recessive	GG *vs.* AA+AG	0.510 (0.106-2.460)	0.401	143.639
Overdominant	AG *vs.* AA+GG	1.452 (0.650-3.246)	0.363	143.610
Additive	G	0.973 (0.530-1.787)	0.929	144.426

ON – Optical Neuritis; OR: odds ratio; CI: confidence interval; *p* value: significance level (alpha = 0.05); AIC: Akaike information criterion; Statistically significant results marked in bold; The most robust genetic model underlined (selected based on the lowest AIC value).

Table S9. Genotype and allele frequencies of *TERF1* rs1545827 and *TERF2* rs251796 within ON and reference groups subjects (age>30).

Gene	Genotype/Allele	ON group (n=40) N (%)	Control group (n=81) N (%)	p-value
***TERF1* (rs1545827)**	CC	10 (25)	27 (33.3)	0.583
	CT	23 (57.5)	39 (48.1)	
	TT	7 (17.5)	15 (18.5)	
	In total:	40 (100)	81 (100)	
	Allele:<br>C<br>T	 43 (53.8)<br>37 (46.2)	 93 (57.4)<br>69 (42.6)	0.589
***TERF2* (rs251796)**	AA	21 (52.5)	42 (51.9)	0.652
	AG	13 (32.5)	31 (38.3)	
	GG	6 (15)	8 (9.9)	
	In total:	40 (100)	81 (100)	
	Allele:<br>A<br>G	 55 (68.8)<br>25 (31.2)	 115 (71)<br>47 (29)	0.720

ON – optic neuritis; *p*-value: significance level (alpha = 0.05).

Table S10. Binary logistic regression analysis within patients with ON and reference group subjects (age>30).

***TERF1* (rs1545827):**				
**Model**	**Genotype/Allele**	**OR (95% CI)**	**p-value**	**AIC**
Codominant	CT *vs.* CC<br>TT *vs.* CC	1.592 (0.654-3.877)<br>1.260 (0.397-3.995)	0.306<br>0.695	156.477
Dominant	CT+TT *vs.* CC	1.500 (0.640-3.516)	0.351	154.676
Recessive	TT *vs.* CC+CT	0.933 (0.347-2.511)	0.891	155.551
Overdominant	CT *vs.* CC+TT	1.457 (0.679-3.126)	0.334	154.630
Additive	T	1.167 (0.673-2.026)	0.582	155.267
***TERF2* (rs251796):**				
**Model**	**Genotype/Allele**	**OR (95% CI)**	**p-value**	**AIC**
Codominant	AG *vs.* AA<br>GG *vs.* AA	0.839 (0.365-1.929)<br>1.500 (0.460-4.887)	0.679<br>0.501	156.735
Dominant	AG+GG *vs.* AA	0.974 (0.456-2.080)	0.946	155.566
Recessive	GG *vs.* AA+AG	1.610 (0.518-5.005)	0.410	154.907
Overdominant	AG *vs.* AA+GG	0.777 (0.349-1.727)	0.535	155.181
Additive	G	1.099 (0.635-1.899)	0.736	155.457

ON – Optical Neuritis; OR: odds ratio; CI: confidence interval; *p* value: significance level (alpha = 0.05); AIC: Akaike information criterion; Statistically significant results marked in bold; The most robust genetic model underlined (selected based on the lowest AIC value).

Table S11. Frequencies of genotypes and alleles of *TERF1* rs1545827 and *TERF2* rs251796 in the long and short telomere groups (T/S median=0.517).

Gene	Genotype/Allele	Long telomeres	Short telomeres	p-value

TERF1 **(rs1545827)**	CC	43 (35)	40 (33.3)	0.806
	CT	66 (53.7)	63 (52.5)	
	TT	14 (11.4)	17 (14.2)	
	In total:	123 (100)	120 (100)	
	Allele:<br>C<br>T	 152 (61.8)<br>94 (38.2)	 143 (59.6)<br>97 (40.4)	0.618
TERF2 **(rs251796)**	AA	65 (52.8)	57 (47.5)	0.573
	AG	47 (38.2)	48 (40)	
	GG	11 (8.9)	15 (12.5)	
	In total:	123 (100)	120 (100)	
	Allele:<br>A<br>G	 177 (72)<br>69 (28)	 162 (67.5)<br>78 (32.5)	0.285

ON – optic neuritis; *p*-value: significance level (alpha = 0.05).

Table S12. Binary logistic regression analysis of *TERF1* rs1545827 and *TERF2* rs251796 in telomere shortening.

***TERF1* (rs1545827):**				
**Model**	**Genotype/Allele**	**OR (95% CI)**	**p-value**	**AIC**
Codominant	CT *vs.* CC<br>TT *vs.* CC	0.975 (0.561-1.692)<br>0.766 (0.335-1.753)	0.927<br>0.528	340.401
Dominant	CT+TT *vs.* CC	0.930 (0.547-1.581)	0.789	338.761
Recessive	TT *vs.* CC+CT	0.778 (0.365-1.659)	0.516	338.409
Overdominant	CT *vs.* CC+TT	1.048 (0.633-1.734)	0.856	338.800
Additive	T	0.901 (0.612-1.327)	0.597	338.553
***TERF2* (rs251796):**				
**Model**	**Genotype/Allele**	**OR (95% CI)**	**p-value**	**AIC**
Codominant	AG *vs.* AA<br>GG *vs.* AA	0.859 (0.502-1.469)<br>0.643 (0.273-1.513)	0.578<br>0.312	339.716
Dominant	AG+GG *vs.* AA	0.807 (0.488-1.336)	0.405	338.138
Recessive	GG *vs.* AA+AG	0.688 (0.302-1.565)	0.372	338.026
Overdominant	AG *vs.* AA+GG	0.928 (0.554-1.553)	0.775	338.751
Additive	G	0.821 (0.564-1.195)	0.303	337.768

ON – Optical Neuritis; OR: odds ratio; CI: confidence interval; *p* value: significance level (alpha = 0.05); AIC: Akaike information criterion; Statistically significant results marked in bold; The most robust genetic model underlined (selected based on the lowest AIC value).

Table S13. Frequencies of genotypes and alleles of *TERF1* rs1545827 and *TERF2* rs251796 in the long and short telomere groups for females (T/S median=0.517).

**Gene**	**Genotype/Allele**	**Long telomeres**	**Short telomeres**	**p-value**

***TERF1* (rs1545827)**	CC	30 (34.1)	28 (33.7)	0.988
	CT	46 (52.3)	43 (51.8)	
	TT	12 (13.6)	12 (14.5)	
	In total:	88 (100)	83 (100)	
	Allele:<br>C<br>T	 106 (60.2)<br>70 (39.8)	 99 (59.6)<br>67 (40.4)	0.911
***TERF2* (rs251796)**	AA	42 (47.7)	44 (53)	0.183
	AG	39 (44.3)	27 (32.5)	
	GG	7 (8)	12 (14.5)	
	In total:	88 (100)	83 (100)	
	Allele:<br>A<br>G	 123 (69.9)<br>53 (30.1)	 115 (69.3)<br>51 (30.7)	0.902

ON – optic neuritis; *p*-value: significance level (alpha = 0.05).

Table S14. Binary logistic regression analysis of *TERF1* rs1545827 and *TERF2* rs251796 in telomere shortening in females.

***TERF1* (rs1545827):**				
**Model**	**Genotype/Allele**	**OR (95% CI)**	**p-value**	**AIC**
Codominant	CT *vs.* CC<br>TT *vs.* CC	0.998 (0.515-1.935)<br>0.933 (0.360-2.417)	0.996<br>0.887	240.886
Dominant	CT+TT *vs.* CC	0.984 (0.522-1.855)	0.961	238.908
Recessive	TT *vs.* CC+CT	0.934 (0.394-2.214)	0.877	238.886
Overdominant	CT *vs.* CC+TT	1.019 (0.559-1.857)	0.951	238.906
Additive	T	0.974 (0.619-1.530)	0.908	238.897
***TERF2* (rs251796):**				
**Model**	**Genotype/Allele**	**OR (95% CI)**	**p-value**	**AIC**
Codominant	AG *vs.* AA<br>GG *vs.* AA	1.513 (0.792-2.892)<br>0.611 (0.220-1.701)	0.210<br>0.346	237.484
Dominant	AG+GG *vs.* AA	1.236 (0.678-2.253)	0.490	238.433
Recessive	GG *vs.* AA+AG	0.511 (0.191-1.369)	0.511	237.067
Overdominant	AG *vs.* AA+GG	1.651 (0.886-3.077)	0.115	236.395
Additive	G	0.974 (0.626-1.515)	0.907	238.896

ON – Optical Neuritis; OR: odds ratio; CI: confidence interval; *p* value: significance level (alpha = 0.05); AIC: Akaike information criterion; Statistically significant results marked in bold; The most robust genetic model underlined (selected based on the lowest AIC value).

Table S15. Frequencies of genotypes and alleles of *TERF1* rs1545827 and *TERF2* rs251796 in the long and short telomere groups for subjects aged≤30 (T/S median=0.517).

**Gene**	**Genotype/Allele**	**Long telomeres 1**	**Short telomeres 0**	**p-value**
***TERF1* (rs1545827)**	CC	25 (36.8)	21 (38.9)	0.787
	CT	37 (54.4)	30 (55.6)	

	TT	6 (8.8)	3 (5.6)	
	In total:	68 (100)	54 (100)	
	Allele:<br>C<br>T	 87 (64)<br>49 (36)	 72 (66.7)<br>36 (33.3)	0.660
***TERF2* (rs251796)**	AA	33 (48.5)	26 (48.1)	0.911
	AG	29 (42.6)	22 (40.7)	
	GG	6 (8.8)	6 (11.1)	
	In total:	68 (100)	54 (100)	
	Allele:<br>A<br>G	 95 (69.9)<br>41 (30.1)	 74 (68.5)<br>34 (31.5)	0.822

ON – optic neuritis; *p*-value: significance level (alpha = 0.05).

Table S16. Binary logistic regression analysis of *TERF1* rs1545827 and *TERF2* rs251796 in telomere shortening for subjects aged≤30.

***TERF1* (rs1545827):**				
**Model**	**Genotype/Allele**	**OR (95% CI)**	**p-value**	**AIC**
Codominant	CT *vs.* CC<br>TT *vs.* CC	1.036 (0.487-2.202)<br>1.680 (0.374-7.548)	0.927<br>0.499	171.028
Dominant	CT+TT *vs.* CC	1.095 (0.524-2.286)	0.810	169.460
Recessive	TT *vs.* CC+CT	1.645 (0.392-6.906)	0.496	169.036
Overdominant	CT *vs.* CC+TT	0.955 (0.466-1.958)	0.900	169.502
Additive	T	1.163 (0.639-2.115)	0.622	169.274
***TERF2* (rs251796):**				
**Model**	**Genotype/Allele**	**OR (95% CI)**	**p-value**	**AIC**
Codominant	AG *vs.* AA<br>GG *vs.* AA	1.039 (0.488-2.211)<br>0.788 (0.227-2.730)	0.922<br>0.707	171.332
Dominant	AG+GG *vs.* AA	0.985 (0.482-2.013)	0.967	169.516
Recessive	GG *vs.* AA+AG	0.774 (0.235-2.552)	0.674	169.341
Overdominant	AG *vs.* AA+GG	1.082 (0.524-2.233)	0.832	169.473
Additive	G	0.940 (0.547-1.617)	0.824	169.468

ON – Optical Neuritis; OR: odds ratio; CI: confidence interval; *p* value: significance level (alpha = 0.05); AIC: Akaike information criterion; Statistically significant results marked in bold; The most robust genetic model underlined (selected based on the lowest AIC value).

Table S17. Frequencies of genotypes and alleles of *TERF1* rs1545827 and *TERF2* rs251796 in the long and short telomere groups for subjects aged>30 (T/S median=0.517).

**Gene**	**Genotype/Allele**	**Long telomeres**	**Short telomeres**	**p-value**
***TERF1* (rs1545827)**	CC	18 (32.7)	19 (28.8)	0.628
	CT	29 (52.7)	33 (50)	
	TT	8 (14.5)	14 (21.2)	
	In total:	55 (100)	66 (100)	

	Allele:<br>C<br>T	65 (59.1)<br>45 (40.9)	71 (53.8)<br>61 (46.2)	0.407
***TERF2* (rs251796)**	AA	32 (58.2)	31 (47)	0.443
	AG	18 (32.7)	26 (39.4)	
	GG	5 (9.1)	9 (13.6)	
	In total:	55 (100)	66 (100)	
	Allele:<br>A<br>G	82 (74.5)<br>28 (25.5)	88 (66.7)<br>44 (33.3)	0.181

ON – optic neuritis; *p*-value: significance level (alpha = 0.05).

Table S18. Binary logistic regression analysis of *TERF1* rs1545827 and *TERF2* rs251796 in telomere shortening for subjects aged>30.

***TERF1* (rs1545827):**				
**Model**	**Genotype/Allele**	**OR (95% CI)**	**p-value**	**AIC**
Codominant	CT *vs.* CC<br>TT *vs.* CC	0.928 (0.411-2.096)<br>0.603 (0.204-1.779)	0.857<br>0.360	169.799
Dominant	CT+TT *vs.* CC	0.831 (0.383-1.804)	0.640	168.521
Recessive	TT *vs.* CC+CT	0.632 (0.244-1.641)	0.346	167.832
Overdominant	CT *vs.* CC+TT	1.115 (0.545-2.283)	0.765	168.651
Additive	T	0.798 (0.472-1.348)	0.399	168.024
***TERF2* (rs251796):**				
**Model**	**Genotype/Allele**	**OR (95% CI)**	**p-value**	**AIC**
Codominant	AG *vs.* AA<br>GG *vs.* AA	0.671 (0.308-1.460)<br>0.538 (0.162-1.786)	0.314<br>0.311	169.104
Dominant	AG+GG *vs.* AA	0.637 (0.309-1.310)	0.220	167.225
Recessive	GG *vs.* AA+AG	0.633 (0.199-2.015)	0.439	168.124
Overdominant	AG *vs.* AA+GG	0.748 (0.354-1.583)	0.448	168.162
Additive	G	0.712 (0.418-1.213)	0.212	167.147

ON – Optical Neuritis; OR: odds ratio; CI: confidence interval; *p* value: significance level (alpha = 0.05); AIC: Akaike information criterion; Statistically significant results marked in bold; The most robust genetic model underlined (selected based on the lowest AIC value).
